# Metformin efficacy and safety for colorectal polyps: a double-blind randomized controlled trial

**DOI:** 10.1186/1471-2407-12-118

**Published:** 2012-03-26

**Authors:** Takuma Higurashi, Hirokazu Takahashi, Hiroki Endo, Kunihiro Hosono, Eiji Yamada, Hidenori Ohkubo, Eiji Sakai, Takashi Uchiyama, Yasuo Hata, Nobutaka Fujisawa, Shiori Uchiyama, Akiko Ezuka, Hajime Nagase, Takaomi Kessoku, Nobuyuki Matsuhashi, Shoji Yamanaka, Yoshiaki Inayama, Satoshi Morita, Atsushi Nakajima

**Affiliations:** 1 Division of Gastroenterology, Yokohama City University School of Medicine, Yokohama, Japan; 2 Department of Gastroenterology, Chigasaki Municipal Hospital, Kanagawa, Japan; 3 Gastroenterology Division, Tokyo Metropolitan Hiroo Hospita, Tokyo, Japan; 4 Department of Gastroenterology, Yokohama Rosai Hospital, Yokohama, Japan; 5 Department of Gastroenterology, Hiratsuka City Hospital, Kanagawa, Japan; 6 Department of Gastroenterology, Kanto Medical Center, NTT East, Tokyo, Japan; 7 Department of Pathology, Yokohama City University, Yokohama, Japan; 8 Department of Biostatistics and Epidemiology, Yokohama City University School of Medicine, Yokohama, Japan; 9 Division of Gastroenterology, Yokohama City University School of Medicine, 3-9 Fuku-ura, Kanazawa-ku, Yokohama 236-0004, Japan

## Abstract

**Background:**

Colorectal cancer is one of the major neoplasms and a leading cause of cancer death worldwide, and new preventive strategies are needed to lower the burden of this disease. Metformin, a biguanide, which is widely used for treating diabetes mellitus, has recently been suggestive to have a suppressive effect on tumorigenesis and cancer cell growth. In a previous study conducted in non-diabetic subjects, we showed that oral short-term low-dose metformin suppressed the development of colorectal aberrant crypt foci (ACF). ACF have been considered as a useful surrogate biomarker of CRC, although the biological significance of these lesions remains controversial. We devised a prospective randomized controlled trial to evaluate the chemopreventive effect of metformin against metachronous colorectal polyps and the safety of this drug in non-diabetic post-polypectomy patients.

**Methods/Design:**

This study is a multi-center, double-blind, placebo-controlled, randomized controlled trial to be conducted in non-diabetic patients with a recent history of undergoing colorectal polypectomy. All adult patients visiting the Yokohama City University hospital or affiliated hospitals for polypectomy shall be recruited for the study. Eligible patients will then be allocated randomly into either one of two groups: the metformin group and the placebo group. Patients in the metformin group shall receive oral metformin at 250 mg per day, and those in the placebo group shall receive an oral placebo tablet. At the end of 1 year of administration of metformin/placebo, colonoscopy will be performed to evaluate the polyp formation.

**Discussion:**

This is the first study proposed to explore the effect of metformin against colorectal polyp formation. Metformin activates AMPK, which inhibits the mammalian target of rapamycin (mTOR) pathway. The mTOR pathway plays an important role in the cellular protein translational machinery and cell proliferation. Patients with type 2 diabetes taking under treatment with metformin have been reported to be at a lower risk of cancer development than those not taking under treatment with metformin. We showed in a previous study that metformin suppressed the formation of human colorectal ACF. We therefore decided to conduct a study to determine whether metformin might suppress the formation of human colorectal polyps.

**Trial registration:**

This trial has been registered in the University hospital Medical Information Network (UMIN) Clinical Trials Registry as UMIN000006254

## Background

Colorectal cancer (CRC) is a major neoplasm worldwide [1], and both its prevalence and mortality have been increasing [2]. Removal of colorectal polyps has been to shown to reduce the risk of future development of colorectal cancer and advanced adenoma [3,4]. On the other hand, patients with polyps (adenomas and/or hyperplastic polyps) constitute a high-risk group for the development of metachronous colorectal polyps and/or CRC [5]. Therefore, a paradigm shift from surveillance for early detection of cancer or adenomas (polypectomy) to new strategies for prevention, including chemoprevention, is needed to lower the burden of this disease. Several large epidemiologic and/or clinical studies have evaluated the possible preventive effects of more than 200 agents, including fiber, calcium, and non-steroidal anti-inflammatory drugs (NSAIDs), including aspirin and selective cyclooxygenase-2 (COX-2) inhibitors, in protecting against CRC development [6]. NSAIDs, especially COX-2 inhibitors, administered either alone or in combination with other agents, have shown the most promise, until date, for CRC risk reduction [4], although reports have revealed an increased risk of serious cardiovascular events associated with the use of COX-2 inhibitors [7,8]. In light of the adverse cardiovascular effects of COX-2 inhibitors and lack of demonstrable efficacy of the other agents that had initially shown promise in this setting, novel drugs that would be both safe and effective for CRC prevention need to be developed. CRC is associated with lifestyle-related diseases, such as diabetes mellitus and obesity [9-12], therefore, we considered that these conditions might represent potential new targets for CRC chemoprevention.

Metformin (1,1-dimethylbiguanide hydrochloride) is a biguanide derivative that has long been used widely for treating diabetes mellitus [13]. It decreases basal glucose output by suppressing gluconeogenesis and glycogenolysis in the liver and increasing glucose uptake by the muscle. Because metformin does not directly stimulate insulin secretion, it is associated with a lower risk of hypoglycemia than other oral antidiabetic drugs [14]. The molecular mechanism involved in the action of metformin is liver kinase B-1dependent activation of AMP-activated protein kinase (AMPK) [15]. Patients with type 2 diabetes under treatment with metformin have been reported to be at a lower risk of cancer development (including CRC) than those not under treatment with metformin [16,17]. This evidence suggests that metformin might be a candidate agent for CRC chemoprevention in diabetic patients. However, since diabetes mellitus itself is a risk factor for cancer, treatment of diabetes mellitus may reduce the risk; therefore, it is still unclear whether the suppressive effect of metformin against CRC may be exerted by the direct chemopreventive effect of the drug or be mediated by its antidiabetic effect. Therefore, we considered that in order to validate the chemopreventive effect of metformin, a clinical trial in nondiabetic patients needs to be conducted.

In previous studies, we demonstrated the chemopreventive effect of metformin in two rodent models (a genetic model and a chemically-induced cancer model) and one human study of colorectal carcinogenesis. We showed that metformin suppressed the development of intestinal polyps in adenomatous polyposis coli (APC^Min/+^) mise, a murine model of familial adenomatous polyposis [18]; furthermore, we showed that metformin suppressed azoxymethane-induced formation of colorectal aberrant crypt foci (ACF) by activating AMPK [19]. Both studies were performed in nondiabetic mice, which suggested the direct chemopreventive potential of metformin per se. In the study conducted on nondiabetic human subjects, we showed that oral low-dose administration of metformin (250 mg per day) suppressed the formation of colorectal ACF and that the drug was safe [20]. ACF are considered as a reliable surrogate biomarker of CRC [21], although their biological significance still remains controversial. Therefore, in CRC chemoprevention trials, in general, the incidence of polyps or of the cancer itself is set as the study endpoint. Although the incidence rate of CRC would be the most reliable endpoint, use of this endpoint would be unsuitable for chemoprevention trials, because of the relatively low occurrence rate of CRC in the general population [22] and the long-term observation period that it would necessitate. Moreover, observation of polyps detected in annual colonoscopies until they grow into cancer would be fraught with ethical problems. Thus, we set the appearance of colorectal polyps as a suitable endpoint for our chemopreventive trial.

Thus, we devised a prospective randomized controlled trial to evaluate the chemopreventive effect of metformin against the development of metachronous colorectal polyps and the safety of this drug in nondiabetic post-polypectomy patients.

This is the first clinical trial of metformin as a chemopreventive agent against metachronous colorectal polyps in humans.

## Methods/Design

### Study design and setting

This study is designed as a multi-center, double-blind, placebo-control, randomized controlled trial to be performed in nondiabetic patients with a recent history of undergoing colorectal polypectomy. The study will take place at the Division of Gastroenterology, Yokohama City University Hospital, and its 5 affiliate hospitals. The coordinating office shall be at Yokohama City University Hospital, with the registration, randomized allocation and data collection to be conducted at this site.

### Ethical considerations and registration

The study protocol is in compliance with the Declaration of Helsinki [23] and the Ethics Guidelines for Clinical Research published by the Ministry of Health, Labour, and Welfare, Japan [24]. We obtained approval for this study from the Ethics committee of Yokohama City University Hospital on July 8^th ^2011. The protocol and informed consent forms were approved by the institutional ethics committee at each of the participating institutions. This trial has been registered in the University hospital Medical Information Network (UMIN) Clinical Trials Registry as UMIN000006254. Written informed consent for participation in the study will be obtained from all the participating patients. The trial results will be reported in conformity with the Consolidated Standards of Reporting Trials (CONSORT) 2010 guidelines [25].

### Eligibility criteria

All adult patients visiting the hospital for polypectomy will be recruited for the study.

The inclusion criteria are as follows:

1) No colorectal polyps present after the polypectomy

2) Age 40 to 80 years as on the date of informed consent

3) Willingness to provide written informed consent

The likelihood of development of colorectal polyps in the young is low and the diagnosis history of polyps in the young is usually related to familial adenomatous polyposis or hereditary non-polyposis colorectal cancer; on the other hand, the elderly have various complications. This was the rationale for our setting the age criterion for inclusion in this study as 40 to 80 years.

The exclusion criteria are as follows:

1) History of diabetes mellitus (use of medication and/or HbA1c over 6.5%)

2) History of regular use (defined as at least once per week) of NSAIDs and/or aspirin

3) History of bowel surgery

4) History of malignant disease (excluding carcinoma in adenoma, carcinoma in situ that has already been resected)

5) History of heart failure, renal failure, liver cirrhosis or chronic hepatic failure

6) History of familial adenomatous polyposis

7) History of hereditary non-polyposis colorectal cancer

8) History of inflammatory bowel disease

9) Pregnancy or possibility of pregnancy

10) Patients judged as inappropriate candidates for the trial by the investigators

### Intervention

All eligible patients will be allocated randomly to one of two groups, the metformin group and the placebo group. Endoscopists, doctors at the follow-up outpatient clinics and patients will be blinded to the allocation. Patients in the metformin group shall receive oral metformin at 250 mg per day, and those in the placebo group shall receive oral placebo tablet. At the end of 1 year of administration of metformin/placebo, colonoscopy will be performed to evaluate the polyp formation.

### Outcome measurements

The primary endpoint shall be the prevalence of colorectal polyps and number of polyps after 1-year's intervention. The endoscopic examinations and polypectomies will be performed using Olympus colonoscopes (model H260AZI). The day before the endoscopy, each patient will be instructed to consume a low-residue diet and shall receive 5 mg of oral sodium picosulfate. On the day of the endoscopy, the patients shall receive 2000 ml of polyethylene glycol (PEG). If the feces are not sufficiently clear, an additional 1000 - 2000 ml of PEG may be given to ensure sufficient bowel cleaning. At the time of the polypectomies, the endoscope shall be inserted into the cecum, and the entire colorectum will be carefully observed as the endoscope is pulled back. If any polyps are detected, polypectomy will be performed. At the end of 1 year of administration of metformin/placebo, the same endoscopists will perform the repeat endoscopic examinations. If a polyp(s) is detected at the repeat colonoscopy (after treatment for one year), a biopsy will be performed. A total of 6 endoscopists from Yokohama City University Hospital and the 5 affiliate hospitals will perform the polypectomies and endoscopic examinations. All procedures will be recorded on DVD, and all the polyps will be photographed. The number of polyps in each patient will first be counted by the operators during the performance of the colonoscopy. To further ensure validity, the number of polyps will be counted again through observation of the recorded DVD by 3 blinded expert endoscopists (H.T, H.E, and E.S). If these expert endoscopists judge the colonoscopic examination as having been inadequate in any case, that case will be excluded. The biopsied polyps will be evaluated by expert pathologists (Y.N and S.Y).

The secondary outcomes are (1) the drug safety; adverse events will be monitored by the doctor at every follow-up visit to the outpatient clinic. Adverse events will be graded according to the National Cancer Institute Common Toxicity Criteria for Adverse Events (NCI-CTCAE), version 4.0. Diarrhea, the most frequent adverse event related to use of metformin, will be watched for spontaneous resolution within a few days and/or managed by antiflatulent drugs. If Grade 3 or more severe adverse events appear, the follow-up doctor shall report it (them) to the coordinating office and the case will be withdrawn from the study at that point; (2) laboratory data (fasting blood glucose, fasting blood insulin, HbA1c, total cholesterol, LDL-cholesterol, blood urea nitrogen (BUN), creatinine); (3) the number of ACF. At the time of the 1-year colonoscopy and polypectomy, the lower rectal region from the middle Houston valve to the dentate line will be washed thoroughly with water, sprayed with 0.25% methylene blue, which would be left to stand for 2 min, then again washed thoroughly with water. The number of rectal ACF will be counted with a magnifying endoscope [21]; (4) physical examination findings (body weight, body mass index (BMI)). Metformin is widely used as an antidiabetic drug that improves insulin resistance. The effect of metformin on insulin resistance and the plasma lipid profile will be evaluated by comparing these parameters measured at the baseline and at 1 year in the metformin group and placebo group. All participants will receive physical examination and laboratory tests at the time of the 1-year endoscopic examination and polypectomy; (5) effects of metformin on the cell-proliferative and apoptotic activities in the rectal epithelium and polyps (if any). Colonic epithelial samples will be obtained from the same trial patients by biopsy at the time of the 1-year colonoscopy and polypectomy. The cell-proliferative activity will be evaluated by staining for the proliferative cell nuclear antigen (PCNA), and the cell-apoptotic activity by the terminal deoxynucleotidyl transferase-mediated dUTP-biotin nick end labeling (TUNEL) method; (6) expression levels of protein (AMPK, mTOR/S6K) in the rectal epithelium and polyps (if any) that are thought to be pharmacological targets of metformin. The expression levels of these proteins shall be determined by western blot analysis.

### Randomization

The investigator shall convey the patient's details to the central registration centre via fax. After an eligibility check, the patients will be randomly assigned to receive metformin or placebo at the central registration centre by a computer program, using a minimization method, with stratification by institute, age, gender and BMI. In this way, the patient assignment will be concealed from the investigator. The randomization center will allocate a numbered treatment pack to each patient, which will contain all the drugs or placebos needed to complete a course of the trial treatment for one patient.

### Drug supply

Metformin will be purchased from Dainippon Sumitomo Parma Co., Ltd. The placebo (250 mg lactose) will be purchased from Kokando Co., Ltd, Toyama, Japan. All trial drugs will be packaged identically and identified only by number. Subjects will be instructed to take one tablet of the trial drug after breakfast every day, and to visit the hospital every 4 weeks for evaluation of the subjective symptoms and for receiving a new supply of medication. Compliance will be monitored by counting the empty drug packages returned by the patients at every visit to the outpatient clinic.

### Sample size estimation

We previously showed that metformin administered at 250 mg/d for 1 month directly suppressed the formation of ACF. In that study, mean number of ACF per patient decreased significantly from 8.78 ± 6.45 (baseline) to 5.11 ± 4.99 (at 1 month, *p = 0.007*) [20]. Based on a similar study, Takayama et al. reported that sulindac administration at 300 mg/d for 2 months to post-polypectomy patients suppressed ACF formation, decreasing the number of ACF from 7.70 ± 4.04 (baseline) to 4.00 ± 2.95 (at 2 months, *p < 0.001*) [26]. From these reports, we estimated that metformin and sulindac may have equivalent effect on suppression of ACF formation. Moreover, from the same study, Takayama et al. reported that in the post-polypectmoy patients who received 2-months' intervention, the number of polyps (hyperplastic polyp and adenoma) at 1 year after the treatment was significantly lower in the sulindac group in comparison with that in the placebo group (26/48 (54.2%) vs. 15/48 (31.3%), *p = 0.025*) [26]. Therefore, we speculated that 1-year's treatment with metformin might yield equivalent suppression of metachronous polyp formation to that with sulindac. To detect the reduction in the number of metachronous polyps in the metformin group using the chi-square test with a two-sided significance level of 5% and a power of 80%, it was found that a sample size of 68 patients per group would be necessary. Assuming a 10% dropout rate, we propose to recruit 75 patients per group, that is, a total of 150 patients.

### Statistical analysis

The prevalence of polyps in each group, the primary endpoint, will be compared between the metformin group and placebo group by the chi-square test. The safety, one of the secondary endpoints, will be similarly compared by the chi-square test. The remaining results in the two groups will be compared by the Mann-Whitney *U *test or Student's *t *test. A P value of < 0.05 shall be regarded as indicative of significance. The analysis will be performed using SPSS, version 17.0 (SPSS Inc., Chicago, Il.).

### Trial steering committee and data monitoring committee

The Trial Steering Committee and Data Monitoring Committee shall be located at the Department of Clinical Research, Yokohama City University School of Medicine. The committee shall consist of three people: Yutaka Natsumeda, M.D., Satoshi Inoue, M.D., and Yukiharu Yamaguchi, Ph.D. The Management Team will monitor the trial progress status and data by face-to-face and/or telephonic contact with each of the six sites every month.

### Study flow

**Figure 1:**
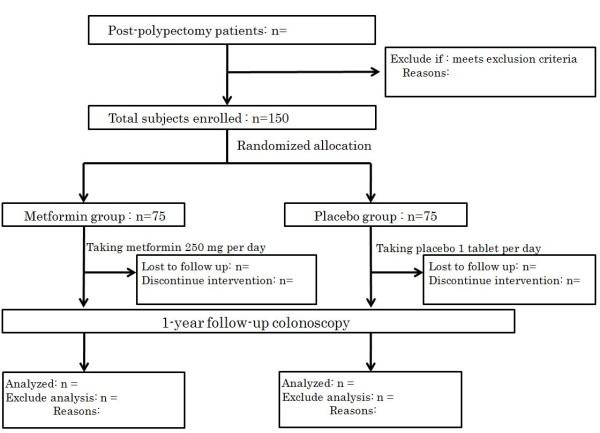
**Study Flow**.

A flow chart of the study is shown in Figure 1.

## Discussion

This is the first study proposed to explore the effect of metformin against colorectal polyp formation. Metformin activates AMPK, which inhibits the mammalian target of rapamycin (mTOR) pathway [15]. The mTOR pathway plays an important role in the cellular protein translational machinery and cell proliferation [27]. The best-characterized downstream effector of mTOR is S6 kinase, which regulates the initiation and elongation phases of translation [28]. Activation of the mTOR pathway has been shown to accelerate cell cycle progression from G1 to S in CRC DLD-1 cells [29]. Therefore, AMPK activation may inhibit cell growth and proliferation by suppressing protein synthesis, thereby having a potent antiproliferative effect. Recent evidence indicates that metformin has a suppressive effect on tumorigenesis and cancer cell growth [30-32]. In one study, metformin was demonstrated to activate AMPK and consequently decrease cellular proliferative activity, to produce a general decrease in protein synthesis in vitro in human breast carcinoma cells [30]. Metformin was also shown to inhibit the proliferation of human prostate cancer cells [32].

This trial may have the following limitations. First, we do not propose to conduct a dose-response study of the effect of metformin on colorectal polyp formation. Until now, trails of metformin for cancer prevention and adjuvant treatment have been conducted using high-dose metformin (500 - 2000 mg per day). However, high-dose metformin use is associated with the risk of development of lactic acidosis and gastrointestinal adverse effects (including diarrhea). Gontier et al. reported from a PET/CT study, that subjects treated with antidiabetic agents, including metformin, showed high and diffuse bowel uptake of 18 F-FDG [33]. This suggests that AMPK is present in abundance in the bowel epithelium and that activation of AMPK by metformin up-regulates the expression of glucose transporters. We showed in a previous study that oral low-dose metformin (250 mg per day) suppressed the formation of human colorectal ACF and was also safe, leading us to surmise that oral low-dose metformin may also be effective for CRC chemoprevention. Therefore, we planned to conduct this trial with low-dose metformin. Second, repeat colonoscopy at 1 year may be too short to allow reliable detection of differences between the groups. However, this is first chemoprevention study of metformin for CRC, long-term administration of placebo to post-polypectomy patients may entail ethical problems. Therefore, this time we planned repeat colonoscopy at 1 year. In order to detect the effect of metformin with less effort, we elected to select participants who had undergone polypectomy for this trial, because these patients constitute a high-risk group for the development of metachronous colorectal polyps and/or CRC [5]. And after safety of chronic metformin administration for non-diabetic patients is confirmed, we would like to follow up the participants of this trial, and conduct long-term chemoprevention trial for CRC.

If metformin were found to be effective for the prevention of CRC, the impact would be extremely large. We consider it of interest, therefore, to determine whether metformin might suppress the formation of human colorectal polyps.

## Abbreviations

CRC: Colorectal cancer; NSAIDs: Nonsteroidal anti-inflammatory drugs; COX-2: Cyclooxygenase-2; ACF: Aberrant crypt foci; AMPK: AMP-activated protein kinase; PCNA: Proliferative cell nuclear antigen; TUNEL: Terminal deoxynucleotidyl transferase-mediated dUTP-biotin nick end labeling.

## Competing interests

The authors declare that they have no competing interests.

## Authors' contributions

TH and AN conceived the study. KH conducted feasibility phase work. HT, TU, NH, US, HK and NM shall perform polypectomy and follow-up colonoscopy. HE, HO and ES will make another count of polyps on DVD record to ensure validity. EY, NH, YH, AE, HN and NM shall recruit participants and follow-up at outpatient clinic. Analysis and interpretation of data is being conducted by SM. SY and YI shall carry out pathological analysis. All authors have read and approve of the final manuscript.

## Pre-publication history

The pre-publication history for this paper can be accessed here:

http://www.biomedcentral.com/1471-2407/12/118/prepub
